# Identification and validation long non-coding RNAs of oral squamous cell carcinoma by bioinformatics method

**DOI:** 10.18632/oncotarget.18178

**Published:** 2017-05-23

**Authors:** Meng Yang, Xingliang Xiong, Longcong Chen, Li Yang, Xian Li

**Affiliations:** ^1^ Research Department, Children Hospital of Chongqing Medical University, Chongqing, China; ^2^ Laboratory of Biomedical Engineering, Chongqing Medical University, Chongqing, China; ^3^ Key Laboratory of Biorheological Science and Technology, Ministry of Education, College of Bioengineering, Chongqing University, Chongqing, China

**Keywords:** oral squamous cell carcinoma, long non-coding RNA, bioinformatics, differential expression analysis, GAS5

## Abstract

Gene markers of oral squamous cell carcinoma (OSCC) have great significance on early diagnosis and treatment of clinical oral cancer. In this study, we used RNA-Seq data from OSCC patients and filtered differentially-expressed long non-coding RNA (lncRNA) to further clarify the molecular mechanism. Firstly, we downloaded datasets of OSCC from National Center for Biotechnology Information(NCBI), which were predicted and analyzed by cufflinks and tophat. Then, differentially expressed lncRNA enrichment was performed with The Database for Annotation, Visualization and Integrated Discovery (DAVID). Finally, we verified the gene expression via *in vitro* assays. Results showed that 52 lncRNAs were significantly differentially expressed compared to those in normal oral tissues, three highly expressed genes (XLOC_002599, XLOC_002634 and XLOC_132858) were verified by RT-PCR, which was consistent with the prediction. XLOC_002634 (GAS5) transcript levels were reduced both *in vivo* and *in vitro* assays, which confirmed that the expression of GAS5 was comparatively low in OSCC. Over-expression of GAS5 in cancer cells inhibited cell proliferation. Moreover, the migration and invasion potential of cancer cells were inhibited compared to control groups. All in all, the study indicated that the decrease in GAS5 expression may contribute to OSCC tumor pathogenesis and serve as a potential target for cancer therapy.

## INTRODUCTION

Every year, 1.6 million people all over the world are diagnosed with squamous cell carcinoma of head and neck (SCCHN) [1]. Oral squamous cell carcinoma (OSCC) is one of the highly aggressive tumors and prone to local recurrence and metastasis [2]. Among these people, one in five died from SCCHN, and half of them were killed by OSCC. OSCC is a complicated process involved a lot of steps, multiple factors and aberrant genes. An increasing number of evidences indicated that various regulators were involved with carcinogenesis. However, the pathogenesis of OSCC was not well understood yet [3]. Recent medical studies confirmed that the etiology of OSCC were associated with DNA deletion, heterozygosity loss and mutation, histone acetylation, gene promoter methylation, proto-oncogene activation and over-expression [4, 5].

Long non-coding RNA (lncRNA), with transcription length between 200 nt to 100 kb, always exist in nucleus or cytoplasm, they do very little or have no encoding proteins by themselves [6]. LncRNA is an important participant in gene expression, their differential expression may possibly affect the corresponding function performance. The role lncRNA played in life activities was still in its infancy, while evidence represented the close relationship between lncRNA and the development of cancer [7]. Genes were expressed differentially between normal cells and cancer cells. Significantly differentially expressed lncRNAs might play an important part in cancer pathogenesis. Some genes promoted carcinogenesis while others inhibited [8]. Next generation sequencing techonology (RNA-seq) [9], as a new technology with high precision and reliability has been applied in screening various tumor genes, such as LncRNA Transforming Growth Factor β(ATB) in breast cancer [10], lncRNA nuclear-enriched abundant transcript 1(NEAT1) in prostate cancer [11].

Growth arrest specific 5 (GAS5) was one of the earliest discovered lncRNAs, and its high expression was firstly discovered in growth inhibition rat of NIH3T3 fiber raw cells [12]. Recent studies indicated that GAS5 has been found low expression in many types of tumors including breast cancer [13], colorectal cancer [14] and prostate cancer [15], however, their functional significance still needs to be established. Breast cancers showed a significantly lower GAS5 expression compared to normal breast epithelial tissues, low expression can induce growth arrest and apoptosis independently of other stimuli in breast cancer cell lines [16]. Mourtada-Maarabouni found that RNA interference GAS5 can protect leukemic and primary human T cells from the Rapamycin anti-proliferative effect [17]. All the evidence suggested that the down-regulation of GAS5 closely related to the development and metasis of cancers, and become a hot spot in cancer research.

In the study, we investigated the characteristics of human genome and used RNA-Seq data from OSCC patients to filter significantly differentially expressed lncRNAs. Relevant functions of differently expressed genes were analyzed by bioinformatic method. As a result, the expression level of GAS5 in OSCC was much lower than that in oral normal tissues, which provided an evidence that some relationship between GAS5 and occurrence of OSCC may exist. GAS5 was then transferred into OSCC cells and its roles were investigated in tumor progression. This study suggested that over-expression of lncRNA GAS5 may function as a therapeutic target for OSCC treatment.

## RESULTS

### Prediction of lncRNA

**Figure 1:**
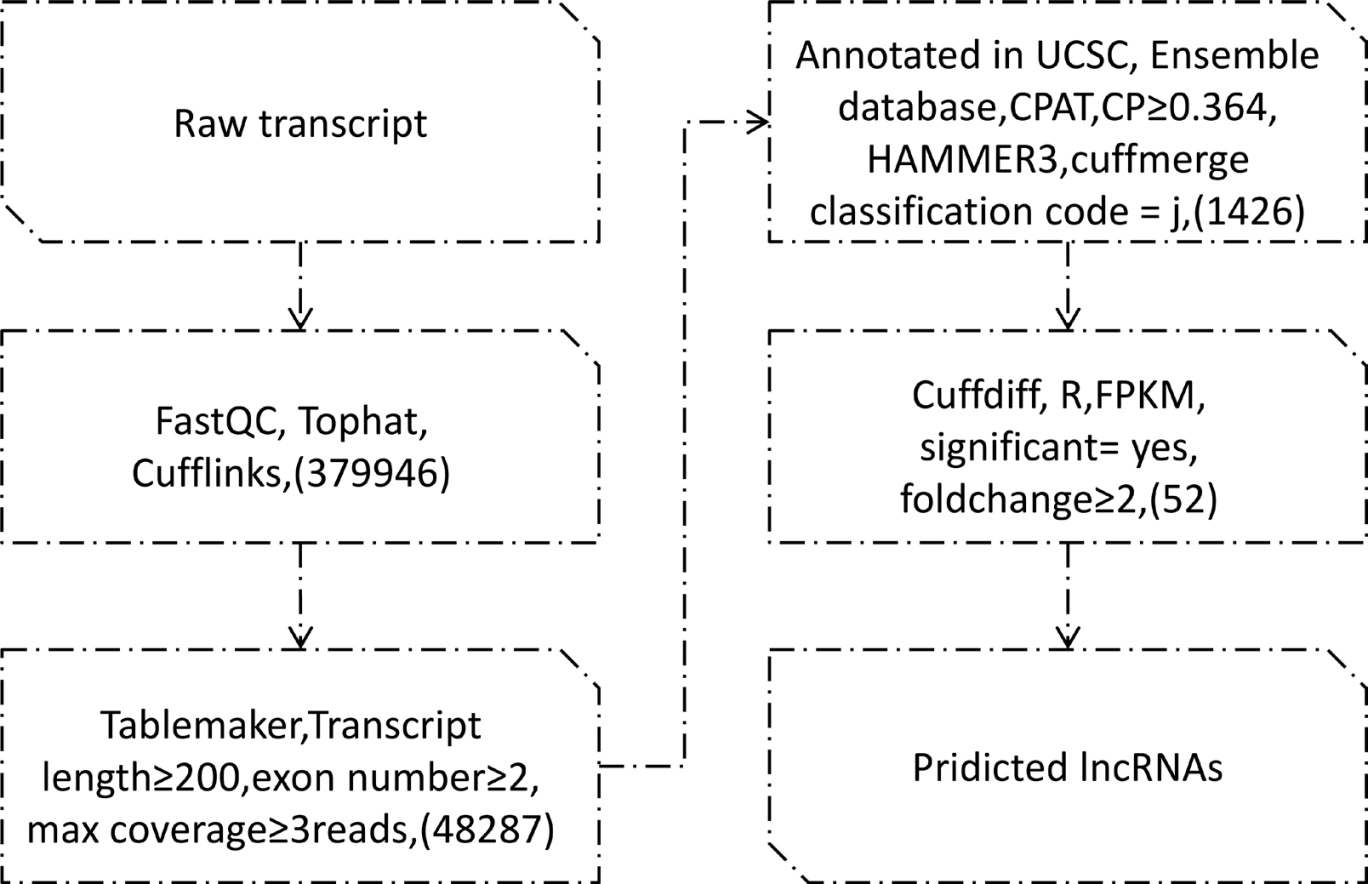
Pipeline for the Identification of OSCC lncRNAs

The data was processed and reconstructed by the transcription group. Results showed that tophat overall read mapping rates were over 99.1%, pair alignment rates were over 79.7%, and 379946 transcripts were assembled by cufflinks. Tablemaker filtered transcripts length, extron number and coverage, and 48287 transcripts left. Known transcripts were screened out and 1426 lncRNAs were obtained through classification code “j” from cuffmerge. Figure 1. showed the filtering process.

### Differential expression analysis

**Figure 2:**
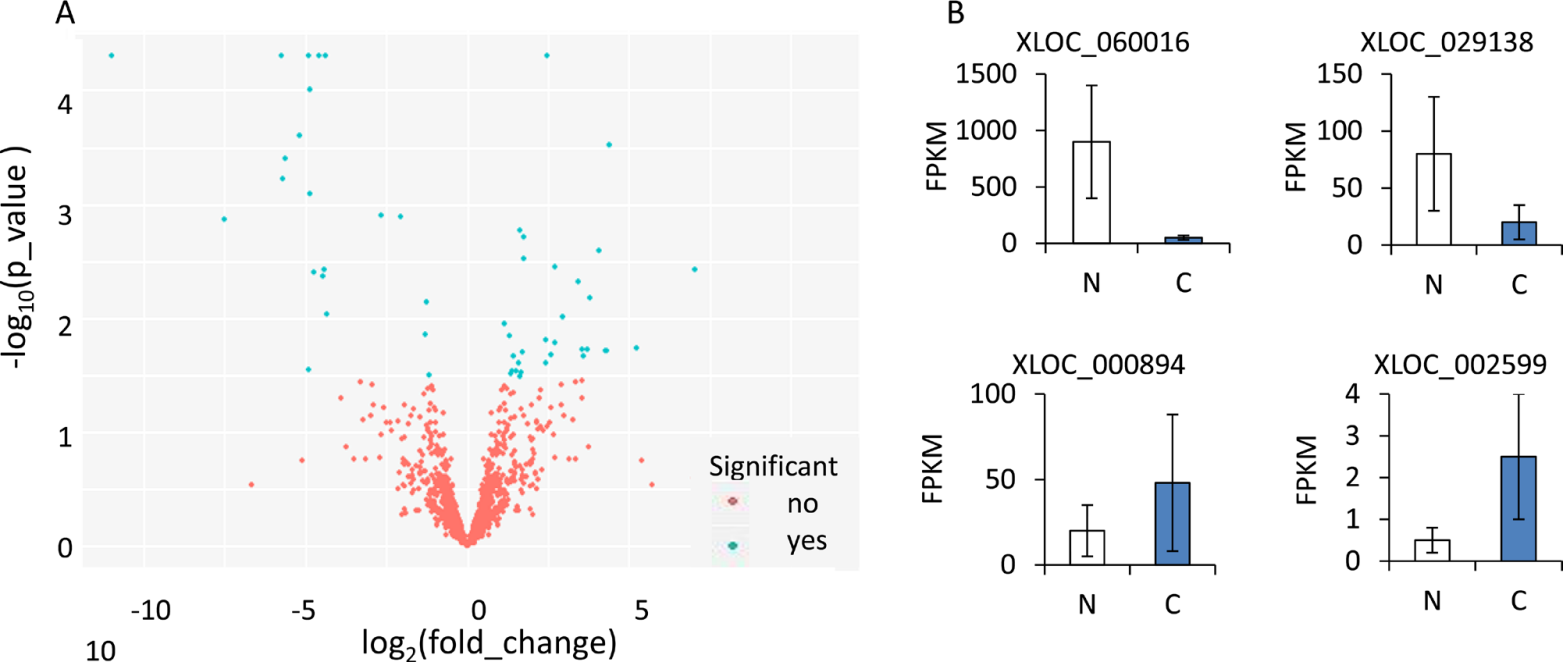
(**A**) Volcano image of lncRNAs with different fold changes of OSCC and Normal tissues. (**B**) FPKM of XLOC_060016, XLOC_029138, XLOC_000894 and XLOC_002599 between OSCC and Normal issues.

52 significant “yes” transcripts left, including 31 up-regulated and 21 down-regulated genes, the log_2_ transformed expression values for differentially expressed lncRNAs were shown in volcano graph (Figure 2A). Four significantly differentially expressed lncRNAs (XLOC_060016, XLOC_029138, XLOC_000894 and XLOC_002599) were measured by FPKM value, Figure 2B demonstrated the different expression of lncRNAs distributed in cancer (C) and under normal (N) conditions.

### Function analysis

**Figure 3:**
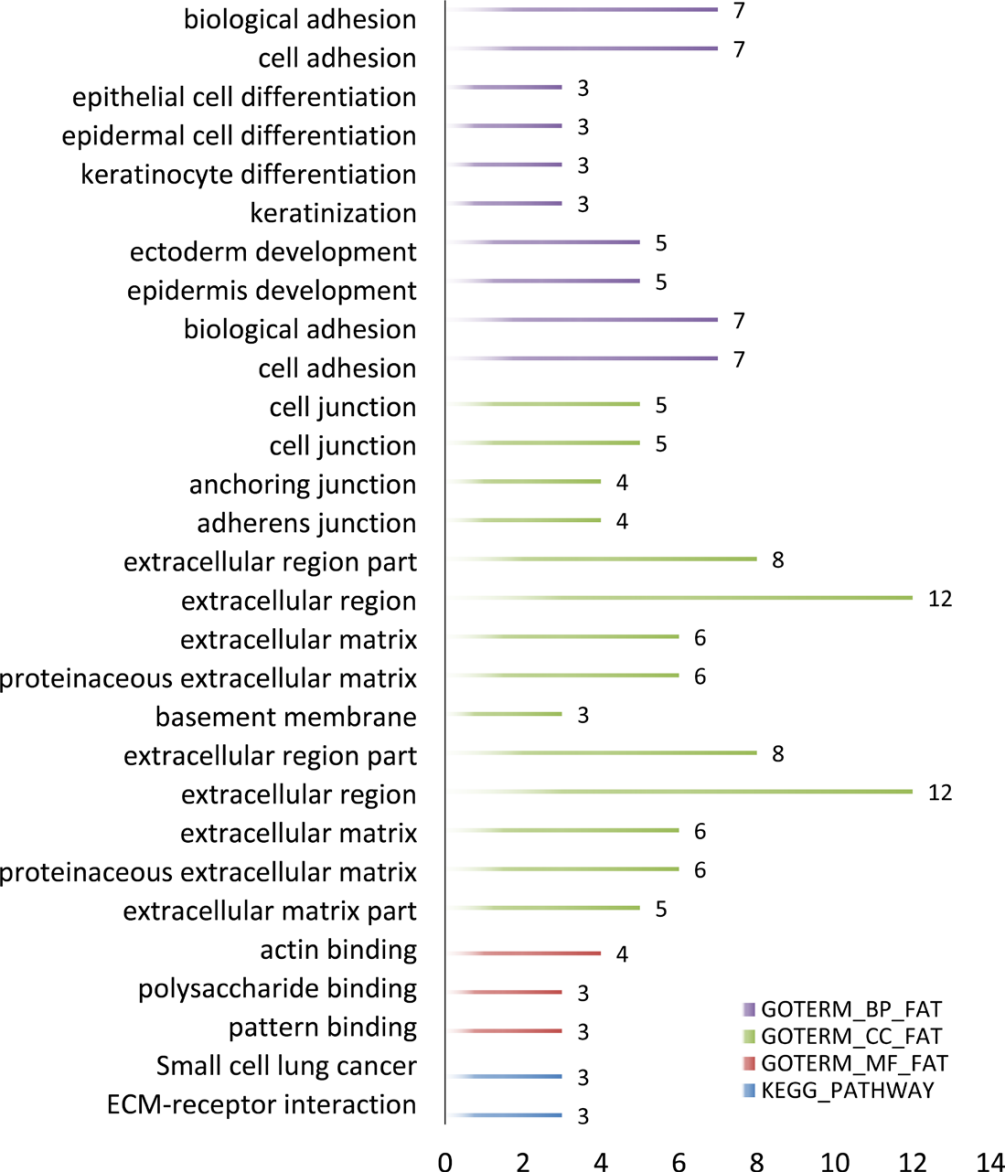
GO term and KEGG pathway of predicted OSCC lncRNAs number

In order to further analyze the differential expression lncRNAs, we performed enrichment analysis of these genes in DAVID. GO-enriched analysis showed that on the biological process (BP), the differential expression genes were associated with biological attachment, cell adhesion, etc.; as to molecular function (MF), the genes were related to actin binding polysaccharide binding and pattern binding; cellular component (CC) was closely correlated with extracellular matrix and cell junction. KEGG pathways of these lncRNAs included small cell lung cancer and ECM-receptor interaction (Figure 3).

### Gene expression by RT-PCR

**Figure 4:**
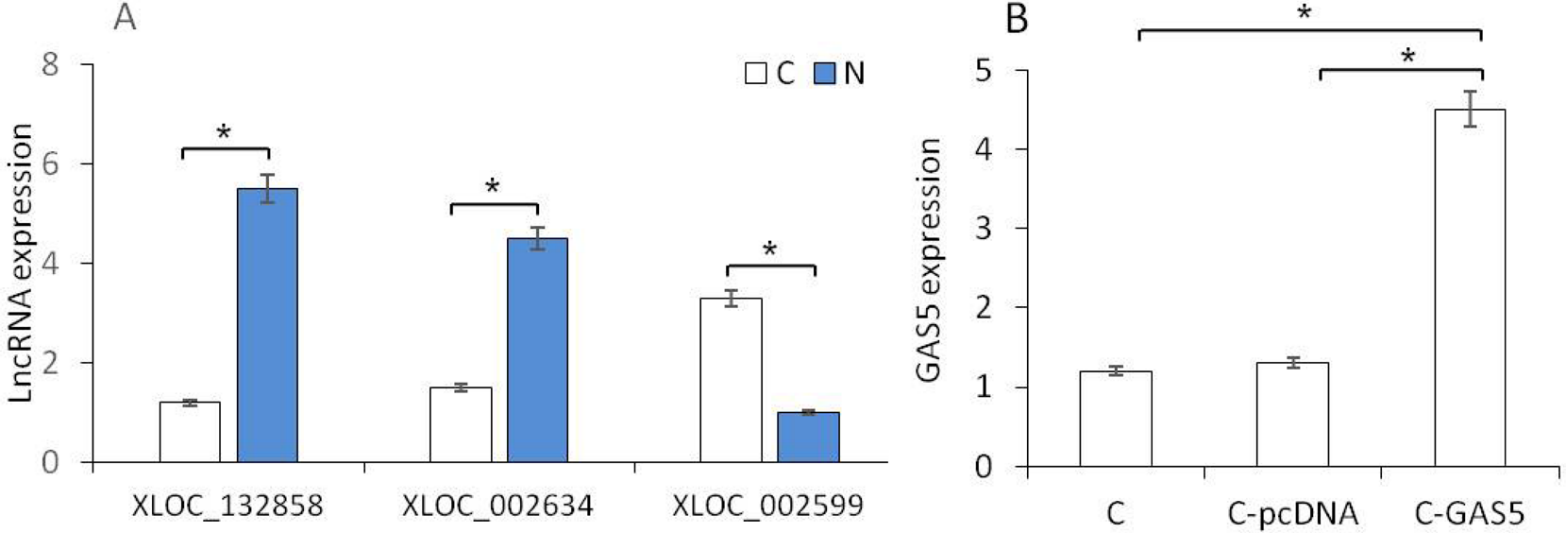
(**A**) RT-PCR analysis of different gene expression between OSCC and Normal tissues. (**B**) GAS5 gene expression in cancer cells, C-pcDNA cells and C-GAS5 cells.(*p* < 0.05).

RT-PCR was used to verify the expression of lncRNAs. Three high-expressed lncRNAs were selected, incuding an up-regulated gene (XLOC_002599) and two down-regulated genes (XLOC_002634 and XLOC_132858). The expression level of these three genes reflected the same results as predicted in the OSCC tissues (Figure 4A). XLOC_002634 (GAS5), as a new gene in OSCC study, presented significantly lower in OSCC samples than that in normal tissues (*p* < 0.05). Therefore, GAS5 was chosen for the further cell experiment. pcDNA3.1-GAS5 was transferred into cancer cell lines and the expression of GAS5 in C-GAS5 group was significantly increased more than that in C-pcDNA group and Cancer group (each, *p* < 0.05, Figure 4B), suggesting the successful pcDNA3.1-GAS5 transfection. C-GAS5 group means cancer cells transfected with pcDNA3.1-GAS, C-pcDNA group means cancer cells transfected with vector pcDNA3.1 only, and C group means cancer cells without any treatment.

### Cell proliferation

**Figure 5:**
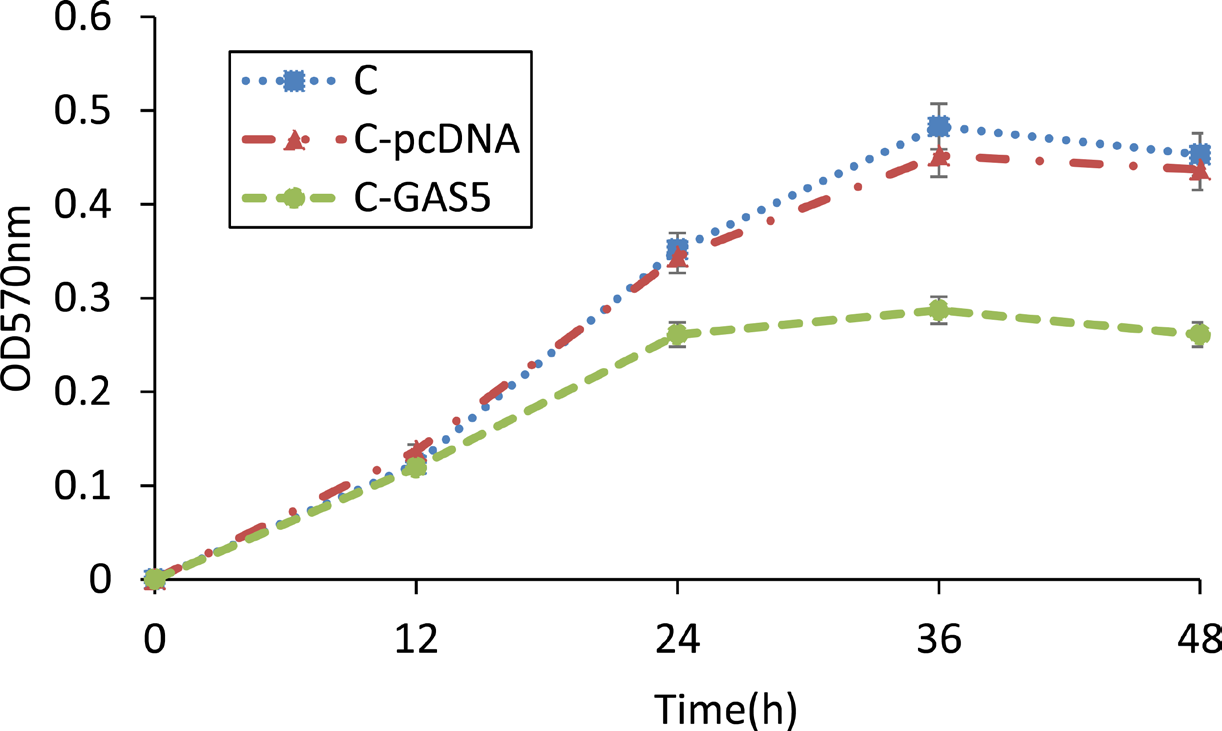
MTT method detected changes of OSCC cell proliferation after transfection of pcDNA-GAS5, 570 nm absorbance of C, C-pcDNA and C-GAS5 from 0 h to 48 h (*p* < 0.05)

MTT colorimetry was used to detect cell survival, NK activity and cell proliferation. Results revealed that the growth of cancer cells was sharply inhibited after transfection with pcDNA3.1-GAS5 for 24 h. Furthermore, the cell survival of C-GAS5 group was much lower than C-pcDNA group and C group after 36 h incubation, and GAS5-transfected group was reduced steadily in the next 12 h (Figure 5). Significant Difference was shown before and after GAS5 transfection experiment. However, there was no difference of the proliferation between C-pcDNA group and C group, indicating that vector pcDNA3.1 may have no effect on the growth of OSCC cells.

### Cell migration

**Figure 6:**
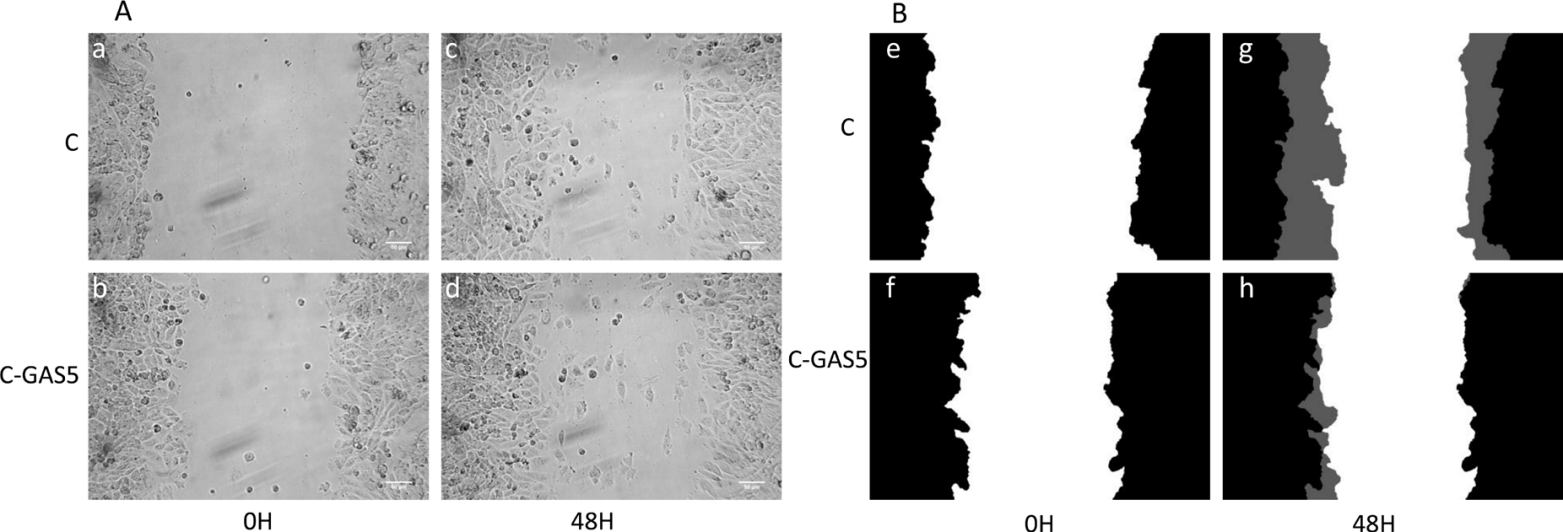
The migration ability of OSCC cells after transfection of GAS5 (**A**) phase contrast image of 0 h (a, b) and 48h (c, d), (**B**) corresponding wound mask image 0 h initial wound mask black (e, f), and 48 h revised wound mask grey (g, h). Image view 50× magnification.

Cell migration ability was detected with wound healing assay. As shown in Figure 6, the wound mask color showed the increased level, for example, black represented initial wound mask, and grey represented the revised area. Cells transfected with pcDNA3.1-GAS5 appeared held-up while non-transfected cancer cells spread extensively and covered larger grey area. These results indicated that overexpressed GAS5 can inhibit the migration of oral cancer cells *in vitro*.

### Cell invasion

**Figure 7:**
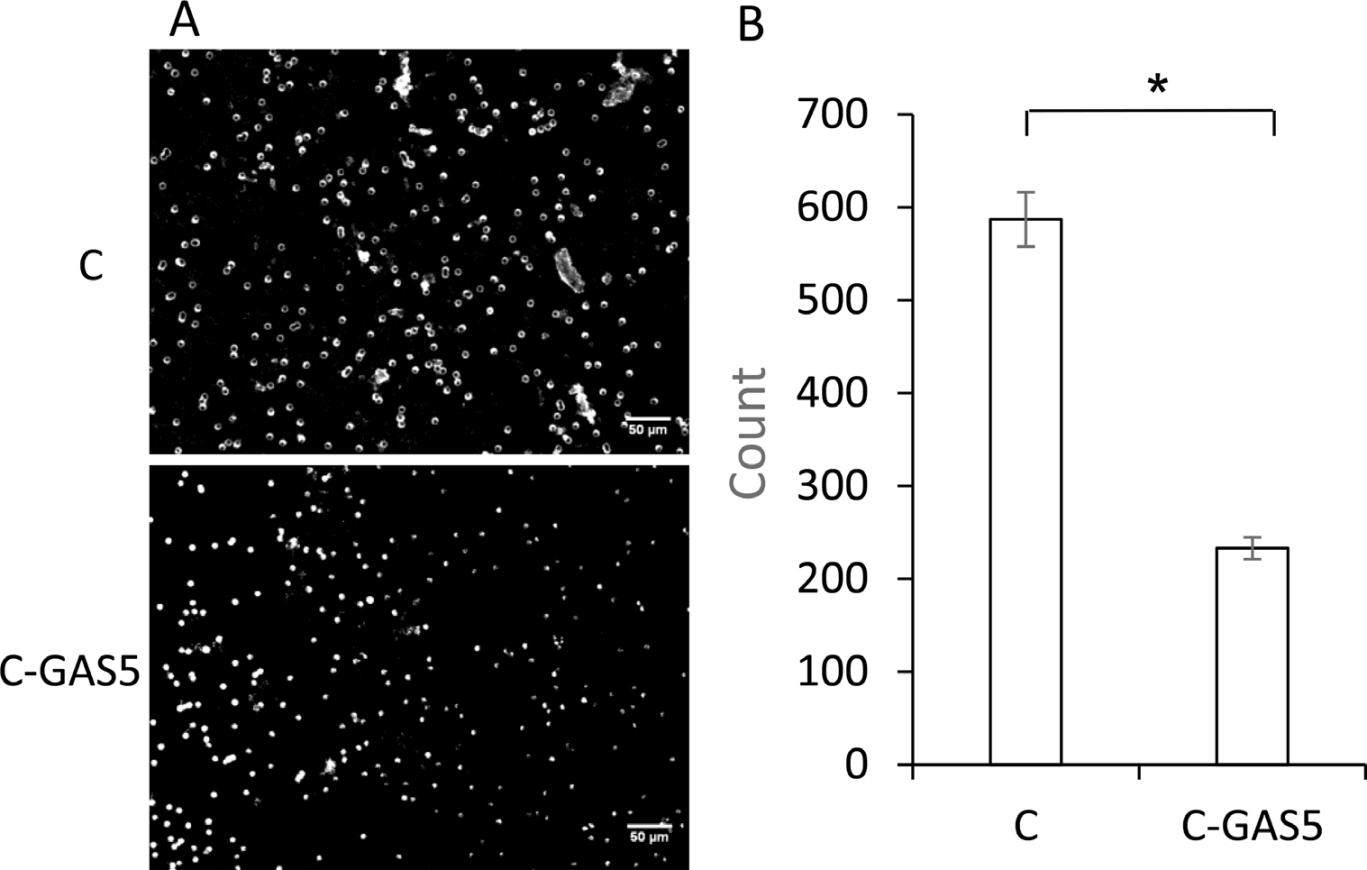
(**A**) Transwell assay of OSCC cell invasion ability after GAS5 transfection for 48 h, image view 50× magnification. (**B**) Statistical analysis count numbers of cancer cells and C-GAS5 cells in the same view (*p* < 0.05).

Meanwhile, invasion ability of cells was detected with Transwell assay. Images were captured at random after 48 h. As shown in Figure 7, the number of C-GAS5 cells was decreased for about half, which showed lower intracellular transport capability after transfection. In other words, the down-regulation of lncRNA GAS5 may possibly be a key of OSCC with high migratory and invasiveness.

## DISCUSSION

Extensive research has revealed that the abnormal expression of lncRNAs may be closely related to tumor and can be used as markers of cancer intestinal diagnosis [18]. In the process of genetic information, lncRNAs play an important role in cell regulatory with gene expression and affect the main cell pathway [19]. Due to various lncRNAs with different regulation mode, predicting and differential expression analysis, lncRNA of OSCC will be of great importance to seek the potential of gene therapy. In this study, we used several bioinformatics tools and screened lncRNAs from OSCC RNA-Seq data. According to differential expression analysis, 52 lncRNAs were found including 31 up-regulated and 21 down-regulated lncRNAs. Functional enrichment analysis and protein-protein interaction network analysis for lncRNAs were carried out with bioinformatics method. The differentially expressed genes were analyzed in DAVID database to determine certain regulatory function, which provided effective information for OSCC basic research and clinical application.

Among these significant differencially expression genes, GAS5 was a common down-regulated genes, and this type of genes was seen as anti-oncogenes, which can affect cell invasion and metastasis in tumor. GAS5 was a new gene for OSCC and chosen as a target gene for validation. RT-PCR results showed that GAS5 expression in OSCC was obviously lower than that in normal tissues. In addition, we transferred GAS5 into OSCC cell lines to increase the gene expression. The OSCC cells were incubated for 48 h and cell proliferation was analyzed using MTT assays, showing that tumor cells were inhibited obviously in C-GAS5 group; wound healing experiment turned out lower cell migration ability and invasion cell number decreased significantly in transwell assay after GAS5 transfection. All of the above results revealed that the over-expression of GAS5 inhibited tumor proliferation, migration and invasion ability, suggesting that the down-regulated expression of GAS5 correlated with OSCC occurrence and development.

In summary, bioinformatics method was used to select lncRNA and applied in the study *in vitro* and *in vivo*. Three significantly different expressed genes were verified by RT-PCR, and cell experiment showed interference of GAS5 expression can inhibit the proliferation and metastasis of tumor. In other words, over-expressed GAS5 can inhibit tumor growth and induce cell apoptosis, which may be regarded as an anti-oncogene for OSCC. It can be also clinically used as a new tumor marker and provided a new target for the treatment of OSCC. However, further clinical research and exploration still needed to seek the molecular mechanism for the probable regulation of OSCC biological behavior.

## MATERIALS AND METHODS

### Prediction of lncRNA

RNA-seq datasets of OSCC in this experiment were obtained from NCBI [20] (https://www.ncbi.nlm.nih.gov/sra/ERR519502/). Two groups contain cancer group C and control group N, with 10 samples separately. These 20 samples were analyzed by Immila and the data quality was controlled with FASTQC [21] to guarantee reliable analysis process in the next steps. The data was firstly mapped to the reference genome (Homo sapiens hg38) using Tophat [22]. Then, Cufflinks were adopted to assemble these alignments sequence and Cuffmerge to combine [23]. After that, table maker was introduced to statistical computations, screening out the transcripts with single extron, transcripts’ extron length larger than 200 bp and coverage no less than 3. The known non-coding protein transcripts were filtered out by comparing the data with human reference genome database such as UCSC and ENSEMBL. Finally, coding potential of left data were explored by CPAT [24], and protein domains were generally analyzed with HAMMER3.

### Differential expression analysis

FPKM was calculated in step cufflinks, here we used cuffdiff procedure to analyse the differentially expressed transcripts. These results were then visualized by R, with CummeRbund package [23], volcano image and barplot of genes were obtained. DAVID [25] database was introduced to realize the enrichment (GO term) [26] and pathway analysis (KEGG pathway) [27].

### RT-PCR validation experiment

OSCC tissues and normal oral epithelium tissues were obtained from Dental Hospital of Chongqing Medical University. The primers were designed using Primer 5 and synthesized by Invitrogen. Primers were designed according to the target genes, and the sequences of the primers were as follows.

**Table:** 

Symbol	Primer F	Primer R
Actin	CGTGCGTGACATTAAGGAGAA	GGAAGGAAGGCTGGAAGAGT
GAS5	CCCCAAGGAAGGATGAGAAT	CGTTACCAGGAGCAGAACCA

### *In vitro* validation experiment

OSCC cell lines were obtained from Chongqing Manuik company and cultured in 10% fetal bovine serum and RPMI1640 medium.

Gene over-expression: Lipofectamine RNAiMaX bought from Invitrogen (Carlsbad, CA) was employed as the cell transfection reagent. Cancer cells were plated without antibiotics, and transfected with 1ng pcDNA3.1-GAS5 as grown to 75–90% confluence. RNA extraction, retro-transcription and qPCR were performed.

MTT assay: Cells were collected after 48h transfection. 0.5% MTT were added into 1×10^6^/ml cell suspension, and cells cultured continuously for 4 h, then dimethyl sulfoxide ( DMSO ) was used to extract crystalline materials. Light absorption value of each hole was obtained with ELISA Reader(OD 570 nm) and repeated four times.

Wound healing assay: Cells were seeded on 6-well plate with transfection protocols. After that, a line was drawn in the middle of the board using a 200 μL pipette tip. Pictures were taken by inverted microscope (×50) at 0–48 h post-wounding, and wound mask was calculated with Image Pro Plus software.

Invasion assay: Matrigel mixed with MEM(1:2) were added in Transwell upper room and reacted to gel for 30 min in 37°C, lower room was filled with 500 μL serum medium. Cells were processed into suspension, 3 × 10^4^ numbers of cells were then added in upper room, discarded the supernatant after 48h and fixed with poly formaldehyde in 3 min, followed with cristal violet staining in 5 min. Different inverted microscope visions (×50) were captured to caculate quantification of invading cells.

### Statistical analyses

All the experimental data were analyzed by SPSS18.0 software, with ANOVA single factor analysis and LSD *t*-test. Measurement data were written in x ± s, and significance was set at *p* < 0.05.

## References

[1] Overgaard J, Hansen HS, Specht L, Overgaard M, Cai G, Andersen E, Bentzen J, Bastholt L, Hansen O, Johansen J (2003). Five compared with six fractions per week of conventional radiotherapy of squamous-cell carcinoma of head and neck: DAHANCA 6&7 randomised controlled trial. Lancet.

[2] Zheng M, Jiang YP, Chen W, Li K, Liu X, Gao SY, Feng H, Wang SS, Jiang J, Ma XR, Cen X, Tang YJ, Chen Y (2015). Snail and Slug collaborate on EMT and tumor metastasis through miR-101-mediated EZH2 axis in oral tongue squamous cell carcinoma. Oncotarget.

[3] Pérez-Sayáns M, Suárez-Peñaranda JM, Pilar GD, Barros-Angueira F, Gándara-Rey JM, García-García A (2011). Hypoxia-inducible factors in OSCC. Cancer Letters.

[4] Chuang JY, Chen PC, Tsao CW, Chang AC, Lein MY, Lin CC, Wang SW, Lin CW, Tang CH (2015). WISP-1, a novel angiogenic regulator of the CCN family, promotes oral squamous cell carcinoma angiogenesis through VEGF-A expression. Oncotarget.

[5] Bose P, Thakur SS, Brockton NT, Klimowicz AC, Kornaga E, Nakoneshny SC, Riabowol KT, Dort JC (2014). Tumor cell apoptosis mediated by cytoplasmic ING1 is associated with improved survival in oral squamous cell carcinoma patients. Oncotarget.

[6] Gupta RA, Shah N, Wang KC, Kim J, Horlings HM, Wong DJ, Tsai MC, Hung T, Argani P, Rinn JL (2010). Long non-coding RNA HOTAIR reprograms chromatin state to promote cancer metastasis. Nature.

[7] Mercer TR, Dinger ME, Mattick JS (2009). Long non-coding RNAs: insights into functions. Nature Reviews Genetics.

[8] Crea F, Watahiki A, Quagliata L, Xue H, Pikor L, Parolia A, Wang Y, Lin D, Lam WL, Farrar WL (2014). Identification of a long non-coding RNA as a novel biomarker and potential therapeutic target for metastatic prostate cancer. Oncotarget.

[9] Wang Z, Gerstein M, Snyder M (2009). RNA-Seq: a revolutionary tool for transcriptomics. Nature Reviews Genetics.

[10] Shi SJ, Wang LJ, Yu B, Li YH, Jin Y, Bai XZ (2015). LncRNA-ATB promotes trastuzumab resistance and invasion-metastasis cascade in breast cancer. Oncotarget.

[11] Chakravarty D, Sboner A, Nair SS, Giannopoulou E, Li R, Hennig S, Mosquera JM, Pauwels J, Park K, Kossai M (2014). The oestrogen receptor alpha-regulated lncRNA NEAT1 is a critical modulator of prostate cancer. Nature Communications.

[12] Coccia EM, Cicala C, Charlesworth A, Ciccarelli C, Rossi GB, Philipson L, Sorrentino V (1992). Regulation and expression of a growth arrest-specific gene (gas5) during growth, differentiation, and development. Molecular & Cellular Biology.

[13] Mourtada-Maarabouni M, Pickard MR, Hedge VL, Farzaneh F, Williams GT (2009). GAS5, a non-protein-coding RNA, controls apoptosis and is downregulated in breast cancer. Oncogene.

[14] Lei Y, Jingjing L, Kenan Z, Qingzhong T, Jin L (2016). A tumor suppressive role of lncRNA GAS5 in human colorectal cancer. Open Life Sciences.

[15] Pickard MR, Mourtada-Maarabouni M, Williams GT (2013). Long non-coding RNA GAS5 regulates apoptosis in prostate cancer cell lines. Biochimica Et Biophysica Acta.

[16] Mourtadamaarabouni M, Pickard MR, Hedge VL, Farzaneh F, Williams GT (2008). GAS5, a non-protein-coding RNA, controls apoptosis and is downregulated in breast cancer. Oncogene.

[17] Mourtadamaarabouni M, Hasan AM, Farzaneh F, Williams GT (2010). Inhibition of human T-cell proliferation by mammalian target of rapamycin (mTOR) antagonists requires noncoding RNA growth-arrest-specific transcript 5 (GAS5). Molecular Pharmacology.

[18] Niu Z, Zhang X, Li W, Ming Z, Zhong Y, Hou Y, Zhang Y, Meng X, Wang W, Deng W, Fan N, Yang S (2016). The role and potential mechanisms of LncRNA-TATDN1 on metastasis and invasion of non-small cell lung cancer. Oncotarget.

[19] Li H, An J, Wu M, Zheng Q, Xin G, Li T, Hu P, Lu D (2015). LncRNA HOTAIR promotes human liver cancer stem cell malignant growth through downregulation of SETD2. Oncotarget.

[20] Edgar R, Domrachev M, Lash AE (2002). Gene Expression Omnibus: NCBI gene expression and hybridization array data repository. Nucleic Acids Research.

[21] Sierro N, Battey JN, Ouadi S, Bovet L, Goepfert S, Bakaher N, Peitsch MC, Ivanov NV (2013). Reference genomes and transcriptomes of Nicotiana sylvestris and Nicotiana tomentosiformis. Genome Biology.

[22] Trapnell C, Pachter L, Salzberg SL (2009). TopHat: discovering splice junctions with RNA-Seq. Bioinformatics.

[23] Trapnell C, Roberts A, Goff L, Pertea G, Kim D, Kelley DR, Pimentel H, Salzberg SL, Rinn JL, Pachter L (2012). Differential gene and transcript expression analysis of RNA-seq experiments with TopHat and Cufflinks. Nature Protocols.

[24] Chu K, Wang L, Chen X, Zhang X, Lu X, Zuo H, Zhou Z, Jia P, Liu S, Qu G (2013). CPAT: Coding-Potential Assessment Tool using an alignment-free logistic regression model. Nucleic Acids Research.

[25] Huang DW, Sherman BT, Lempicki RA (2009). Systematic and integrative analysis of large gene lists using DAVID bioinformatics resources. Nature Protocol.

[26] Harris MA, Clark J, Ireland A, Lomax J, Ashburner M, Foulger R, Eilbeck K, Lewis S, Marshall B, Mungall C (2004). The Gene Ontology (GO) database and informatics resource. Nucleic Acids Research.

[27] Kanehisa M, Goto S, Sato Y, Furumichi M, Tanabe M (2012). KEGG for Integration and Interpretation of Large-Scale Molecular Data Sets. Nucleic Acids Research.

